# Incidence and demographics of giant cell tumor of bone in The Netherlands: First nationwide Pathology Registry Study

**DOI:** 10.1080/17453674.2018.1490987

**Published:** 2018-07-10

**Authors:** Arie J Verschoor, Judith V M G Bovée, Monique J L Mastboom, P D Sander Dijkstra, Michiel A J Van De Sande, Hans Gelderblom

**Affiliations:** a Department of Medical Oncology, Leiden University Medical Center, Leiden;; b Department of Pathology, Leiden University Medical Center, Leiden;; c Department of Orthopedic Surgery, Leiden University Medical Center, Leiden, The Netherlands

## Abstract

Background and purpose — Giant cell tumors of bone (GCT-B) are rare, locally aggressive tumors characterized by an abundance of giant cells. Incidence studies for GCT-B are rare. This is the first study using a fully automated 100% covering pathology database, the nationwide Dutch Pathology Registry (17 million inhabitants), PALGA, to calculate incidence rates for GCT-B.

Patients and methods — From PALGA, all pathology excerpts were retrieved for patients diagnosed with GCT-B, giant cell tumors of tenosynovium, and giant cell tumors of soft tissue between January 1, 2009 and December 31, 2013. The incidence of GCT-B was calculated.

Results — In total, 8,156 excerpts of 5,922 patients were retrieved; these included 138 first GCT-B diagnosis. For GCT-B the incidence was 1.7 per million inhabitants per year with a male to female ratio of 1:1.38 and a median age of 35 years (9–77). Most common localization was the femur (35%), followed by the tibia (18%). No differences in localization according to age and sex were found. The incidence rate of local recurrence was 0.40 per million inhabitants per year.

Interpretation — This is the first nationwide study reporting the incidence of GCT-B, based on a nationwide pathology database with 100% coverage of pathology departments. Current incidence calculations are based only on doctor-driven registries. We confirmed that GCT-B is a rare disease with an incidence that is slightly higher than previously published. The relatively young median age of patients and the high incidence of recurrence stresses the importance of developing more effective treatments for this disease.

Giant cell tumor of bone (GCT-B) is a locally aggressive neoplasm composed of sheets of mononuclear cells admixed with uniformly distributed large osteoclast-like giant cells, primarily affecting the metaphysis of long bones (Athanasou et al. 2013). These cells express receptors of nuclear factor kappa-B ligand (RANKL) (Atkins et al. 2000, Roux et al. 2002). GCT-B are rare; however, the incidence is not exactly known and is for example not stated in the World Health Organization (WHO) classification of Tumors of Soft Tissue and Bone (Athanasou et al. 2013). The incidence was recently estimated at between 1.03 and 1.33 per million per year based on cancer registries in Australia, Japan and Sweden (Table 1) (Liede et al. 2014, Amelio et al. 2016). Median age of onset ranges between 20 and 40 years with an equal distribution between the sexes or a slight female predominance (Athanasou et al. 2013, Amelio et al. 2016).

**Table 1.** Review of all available incidence data on GCT-B

Article	Country	Type	Incidence per million inhabitants	Age median (range)	Percentage men
Liede et al. 2014**^a^**	Sweden, Australia, Japan	Doctor-driven	1.03–1.33	20–40 (na)	na
Amelio et al. 2016**^a^**	Sweden	Doctor-driven	1.3	34 (10–88)	48
Current study	The Netherlands	Nationwide pathology registry	1.66	35 (9–77)	42

na: not available. The study by Liede et al. does not report an exact median age, but only a median age group.

**^a^**These studies probably also included patients with a giant cell tumor of the small bones of hands or feet and patients with central giant cell granulomas of the jaw.

Patients with GCT-B typically present with pain, swelling, and often decreased joint movement. In 5–30% of patients a pathologic fracture is noted (Athanasou et al. 2013, van der Heijden 2014b). Although this tumor rarely metastasizes, it is known to be locally aggressive, which may result in joint destruction and, uncommonly, neurological deficit in axial tumors (Athanasou et al 2013). Treatment options are curettage, curettage with an adjuvant treatment, or resection with joint replacement (van der Heijden 2014a). In GCT-B the local recurrence rate is 6–42% (Balke et al. 2008, van der Heijden 2014b). Recently, denosumab, a human IgG2 monoclonal antibody against RANKL, was registered for use in GCT-B and showed tumor response in 2 phase II studies (Thomas et al. 2010, Chawla et al. 2013).

According to our knowledge, current literature on GCT-B contains incidence calculations based solely on cancer registry studies. These studies are doctor-driven with a risk of underreporting (Table 1) (Liede et al. 2014, Amelio et al. 2016). In this study we use the non-profit nationwide network and registry of histo- and cytopathology in the Netherlands, PALGA. This fully automated nationwide database contains all pathology reports in the Netherlands (17 million inhabitants) (Casparie et al. 2007). In an effort not to miss GCT-B cases, our search included the following giant cell containing tumors: GCT-B, tenosynovial giant cell tumors, and giant cell tumors of soft tissue.

We calculated the incidence, demographics, and localizations of GCT-B in a nationwide pathology database study between January 1, 2009 and December 31, 2013.  

## Patients and methods

### Patients

PALGA covers all pathology reports of all pathology laboratories in the Netherlands since 1993 (Casparie et al. 2007). Patient registration in PALGA is based on social service number and thereby multiple reports of one patient will be grouped and not lead to double registration of one patient. Excerpts matching our search criteria were retrieved from PALGA, encoded either as giant cell tumor of bone (PALGA code m9250*) or as giant cell tumor of tenosynovium (m9252*) or pigmented villonodular synovitis (m9252*) or giant cell tumor of soft tissue (m9251*) and terms separately used as free text between January 1, 2009 and December 31, 2013 (Casparie et al. 2007). In our search, giant cell tumors of soft tissue and tenosynovium were included, to be as comprehensive as possible. Additionally, for all these patients historical excerpts were retrieved matching our search criteria. When date of first diagnosis met our 5-year timeframe, the patient was included for incidence calculations. Patients with a giant cell tumor of the small bones of the hands or feet were excluded, since these are considered a separate entity according to the current WHO classification of tumors of soft tissue and bone (Forsyth and Jundt 2013). Patients with a GCT-B affecting the mandible were also excluded, because these are probably central giant cell granulomas of the jaw (Jaffe 1953).

Based on the combination of the historical and current excerpts we could calculate the incidence of first local recurrences during the 5-year study period. It is essential to note that this is not the same as the incidence of recurrences for those patients diagnosed during these 5 years of study. To calculate the latter, a longer interval between the study period and the moment of reporting would be necessary.

Excerpts contained an encrypted patient identification number (allowing for identification of multiple excerpts of one patient), data on age and sex, date of arrival of the histological tissue, and the conclusion of the pathology report. AJV extracted the data and uncertain pathology conclusions in the reports were discussed with HG and JVMGB.

Disaggregated incidence rate calculations for localized and diffuse type are necessary in giant cell tumors of tenosynovium. The PALGA database lacks information on tumor type (i.e., whether a giant cell tumor of tenosynovium is a localized type or a diffuse type as this is a combined diagnosis of radiological and pathological examinations), therefore additional chart review in giant cell tumors of tenosynovium was performed and published elsewhere (Mastboom et al. 2017).

### Data collection

Anonymized data were collected on age, sex, year of diagnosis, localization, GCT type, and date of local recurrence.

### Statistics

For statistical analysis, the Statistical Package for the Social Sciences (SPSS) version 23.0.0 (IBM Corp, Armonk, NY, USA) was used. Incidence of GCT-Bs was calculated per million inhabitants per year and standardized for 5-year age groups and sex for the Dutch population in 2012, as published by the Central Bureau of Statistics (CBS) (Statistics Netherlands 2014). Incidence standardized to the WHO standard population for 5-year age groups was also calculated (Ahmad et al. 2001).

We estimated first recurrences, defined as biopsied lesions or surgically treated recurrences, as the registry contained only reports for histological specimens. Both 95% confidence intervals (CI) for incidence rates (Mid-P exact) and frequencies (Wilson score) were calculated using www.openepi.com.

### Ethics, funding, and potential conflicts of interest

As pathology excerpts were fully anonymized, no ethics approval was necessary for this study. This work was supported by Daiichi-Sankyo with an unconditional financial grant. There are no potential conflicts of interest.  

## Results

### Search results

**Figure 1.**
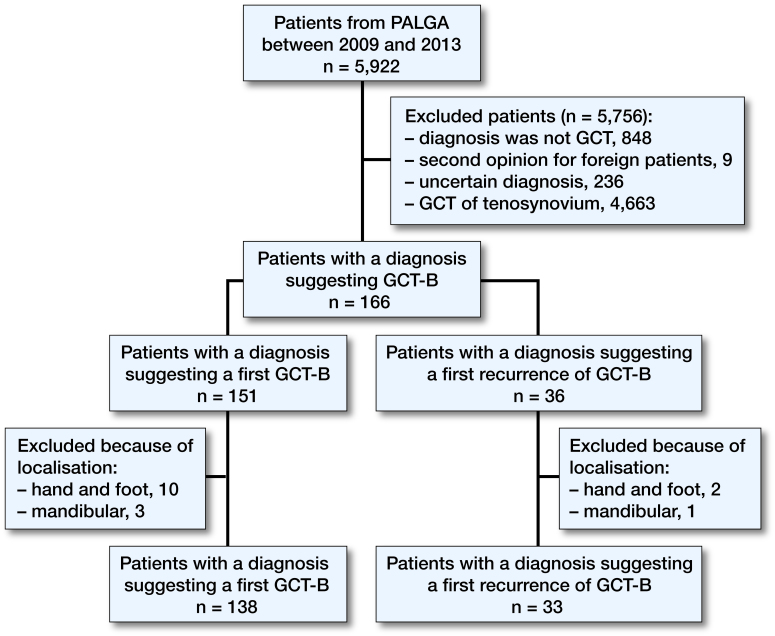
Diagram showing inclusion and exclusion of all patients with GCT in the Netherlands between 2009 and 2013.

From PALGA, 8,156 excerpts of 5,922 patients were retrieved matching the search criteria (Figure 1). Of these 5,922 patients, 5,756 patients were excluded. 151 new cases of GCT-B were identified; however, 13 of these new cases were actually not GCT-B, but either giant cell tumors of the small bones of hands or feet or central giant cell granulomas of the jaw. 15 patients were only diagnosed with a first recurrence during the study period and had a primary tumor before the study period.

**Figure 2.**
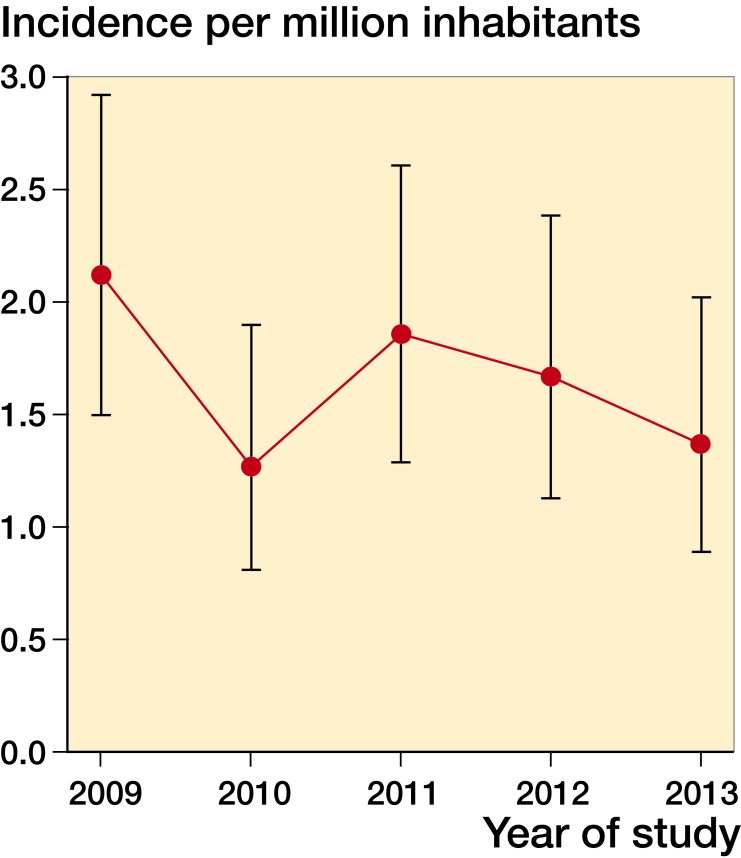
Crude incidence rates of GCT-B in the Netherlands with 95% confidence interval.

**Figure 3.**
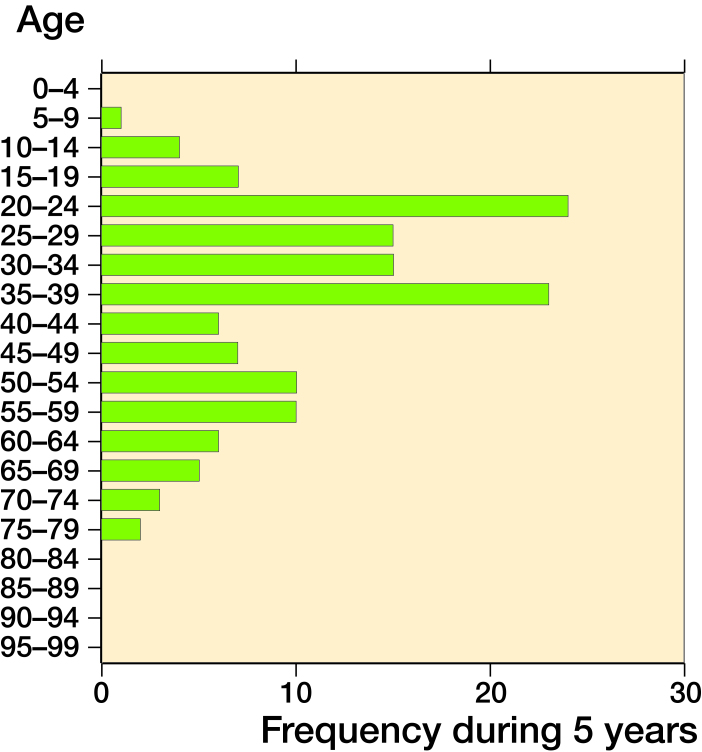
Age distribution of GCT-B in the Netherlands.

This resulted in 138 cases with a crude incidence ranging from 1.3 to 2.1 per million inhabitants per year (mean: 1.7; standard deviation (SD) 0.3; CI 1.4–2.0), age and sex corrected incidence ranged between 1.3 and 2.1 per million inhabitants per year (mean: 1.7; SD 0.3; CI 1.4–1.9), and the WHO standardized incidence was 1.4 to 2.3 per million inhabitants per year (mean: 1.7; SD 0.3; CI 1.4–2.0. Table 2 and Figure 2). 42% of patients were male (CI 34–50). The median age of patients was 35 years (9–77). 6% were below 18 years of age. The age distribution of GCT-B seems to be bimodal with a peak incidence between 20 and 39 and between 50 and 59 years (Figure 3). Most affected localization was the femur (35%), followed by the tibia (18%) (Table 3). During these 5 years, 33 patients were diagnosed with a first recurrence. Consequently, crude incidence of pathology confirmed first recurrence was 0.40 per million inhabitants per year (CI 0.28–0.55). Median age of patients with a first recurrence was 32 years (10–63). Median time between first diagnosis and first recurrence was 23 months (range 2–142). 30% of the recurrences occurred within 1 year, 24% in the second year, and 30% in the third year after diagnosis. Most common localization of recurrence were the femur and tibia (both 30%; Table 4).

**Table 2.** Overview of incidence rates

	Crude incidence per million inhabitants per year (CI)	Age median (range)	Percentage men (CI)
GCT-B total	1.7 (1.4–1.9)	35 (9–77)	42 (34–50)
Long bones	1.3 (1.1–1.6)	35 (9–77)	41 (33–51)
Axial	0.35 (0.21–0.45)	38 (17–73)	46 (29–65)

**Table 3.** Frequencies of localizations of first GCT-B

Localization	Absolute frequency	Percentage (CI)
Femur	48	35 (27–43)
Tibia	25	18 (12–25)
Radius	14	10 (6–16)
Fibula	13	9 (6–15)
Spine	13	9 (6–15)
Ulna	5	4 (2–8)
Pelvis	5	4 (2–8)
Humerus	2	1 (0–5)
Mastoid	2	1 (0–5)
Patella	2	1 (0–5)
Scapula	2	1 (0–5)
Other *	3	2 (1–6)
Unknown	4	3 (1–7)
Total	138	100

**^a^** Other: 4th rib, maxilla and petrous bone.

**Table 4.** Localization of recurrences

Localization	Absolute frequency	Percentage (CI)
Femur	10	30 (17–47)
Tibia	10	30 (17–47)
Spine	4	12 (5–27)
Radius	3	9 (3–24)
Humerus	2	6 (2–20)
Fibula	1	3 (1–15)
Ulna	1	3 (1–15)
Scapula	1	3 (1–15)
Pelvis	1	3 (1–15)
Total	33	100

Incidence rates for the long bones and the axial skeleton were 1.3 (CI 1.1–1.6) and 0.31 (CI 0.21–0.45) per million inhabitants per year, respectively (1 patient could not be allocated to one of the groups). WHO standardized incidence rates were 1.40 and 0.31 per million per year. Incidence of recurrence were 0.32 (CI 0.21–0.47) and 0.07 (CI 0.03–0.15) per million per year. The median age of patients for the 2 groups was 35 (9–77) and 38 (17–73) years. Percentage of males was 41% (CI 33–51) and 46% (CI 29–65) respectively.

The localization of the tumors did not differ according to sex. Only 1 of the patients below 18 years of age had an axial localization of his GCT-B. However, this difference could be attributed to the low incidence of axial GCT-B.

During the 5-year study period, only 1 malignant GCT-B was reported.

## Discussion

This is the first study on GCT-B incidence, based on a fully automated pathology database covering 100% of pathology reports in the Netherlands. Our calculated GCT-B incidence shows a higher number, compared with previously reported incidence rates (Table 1) (Liede et al. 2014, Amelio et al. 2016). In addition, the study by Amelio et al. does not seem to exclude giant cell tumors of the small bones of the hands or feet and central giant cell granulomas of the jaw, suggesting that the actual incidence of giant cell tumors of bone in this study is actually lower (Amelio et al. 2016). However, this is not exactly stated in the paper, but derived from the graphs showing localizations. For the study by Liede et al. (2014), no data on localization were reported. The higher incidence may be explained by the use of the 100% covering Dutch nationwide pathology database PALGA. Incidence of GCT-B seems to decrease slightly during the 5 years of study; this could be attributed to a normal variation in the low absolute count of GCT-B per year. As expected, most GCT-Bs were localized in the lower, weight-bearing extremities, as described in previous studies (Liede et al. 2014, Amelio et al. 2016). Both slight female preponderance and age distribution are comparable to these earlier reports (Liede et al. 2014, Amelio et al. 2016). Age distribution seems to be bimodal (between 20 and 39 years and 50 and 59 years of age), which is comparable to data described by Liede et al. (2014). However, this variation could also be due to the small number.

GCT-Bs generally affect young patients (median age 35 years) and 6% are younger than 18 years. This is lower compared with the 14% in the Swedish study and the approximately 8% in Japan (manually calculated, based on published data) (Liede et al. 2014, Amelio et al. 2016). Reporting bias could be the cause of the higher percentage of patients <18 years with a GCT-B in the Swedish and Japanese registries, because these are doctor-driven registries.

Although we do not calculate an exact incidence rate of first local recurrences for the patients diagnosed between 2009 and 2013 in this study, we calculated an incidence of all first recurrences during this time period of 0.40 per million inhabitants per year. This results in a rate of recurrence of approximately 24% (although the denominator is not exactly known), which is lower compared with the Swedish study (recurrence rate 41%) (Liede et al. 2014, Amelio et al. 2016). 2 retrospective cohort studies reported rates of recurrence between 6 and 42% (Balke et al. 2008, van der Heijden 2014b). The relatively higher recurrence rate in the Swedish study could be attributed to an effect of reporting bias (patients with a recurrence will have a higher chance of being registered). The differences in recurrence could be caused by different treatment strategies, which cannot be calculated in these studies due to a lack of data in our study and the other studies (van der Heijden 2014b).

Compared with the other studies, the reported incidence of malignant GCT-B (1 patient in 5 years, 1 of 138 patients) during these years was much lower than in the study by Liede et al. (27/337). We have no explanation for this difference.

In the future, additional nationwide studies are needed to calculate a more accurate worldwide incidence, because at the moment incidence rates only for countries in North and West Europe are available. Furthermore, the incidence calculations should include information on the incidence of GCT-B subdivided into long bones and the axial skeleton.

In summary, this study is the first to report incidence of GCT-B based on a 100% coverage nationwide pathology database. These incidence numbers are of value for research and healthcare planning.

AJV, JVMGB, HG designed the study. AJV did the data collection and primary analysis of the data. All authors interpreted the data. AJV wrote the manuscript. All authors critically reviewed the manuscript and approved the final version.

*Acta* thanks Peter Holmberg Jørgensen and Claus Lindkaer Jensen for help with peer review of this study.
